# Exploring natural genetic variation in tomato sucrose synthases on the basis of increased kinetic properties

**DOI:** 10.1371/journal.pone.0206636

**Published:** 2018-10-29

**Authors:** Quy-Dung Dinh, Richard Finkers, Adrie H. Westphal, Walter M. A. M. van Dongen, Richard G. F. Visser, Luisa M. Trindade

**Affiliations:** 1 Plant Breeding, Wageningen University and Research, AJ Wageningen, The Netherlands; 2 Graduate School Experimental Plant Sciences, Wageningen University, Wageningen, The Netherlands; 3 Laboratory of Biochemistry, Wageningen University & Research, WE Wageningen, The Netherlands; University of Tsukuba, JAPAN

## Abstract

Sucrose synthase (SuSy) is one key enzyme directly hydrolyzing sucrose to supply substrates for plant metabolism, and is considered to be a biomarker for plant sink strength. Improvement in plant sink strength could lead to enhanced plant growth and yield. Cultivated tomatoes are known to have a narrow genetic diversity, which hampers further breeding for novel and improved traits in new cultivars. In this study, we observed limited genetic variation in *SuSy1*, *SuSy3* and *SuSy4* in 53 accessions of cultivated tomato and landraces, but identified a wealth of genetic diversity in 32 accessions of related wild species. The variation in the deduced amino acid sequences was grouped into 23, 22, and 17 distinct haplotypes for SuSy1/3/4, respectively. Strikingly, all known substrate binding sites were highly conserved, as well as most of the phosphorylation sites except in SuSy1. Two SuSy1 and three SuSy3 protein variants were heterologously expressed to study the effect of the amino acid changes on enzyme kinetic properties, i.e. maximal sucrose hydrolyzing capacity (V_max_), affinity for sucrose (K_m_), and catalytic efficiency (V_max_/K_m_) at 25°C and 16°C. SuSy1-haplotype#3 containing phosphorylation site Ser-16 did not have an improvement in the kinetic properties compared to the reference SuSy1-haplotype#1 containing Arg-16. Meanwhile SuSy3-haplotype#9 from a wild accession, containing four amino acid changes S53A, S106I, E727D and K741E, showed an increase in V_max_/K_m_ at 16°C compared to the reference SuSy3-haplotype#1. This study demonstrates that SuSy kinetic properties can be enhanced by exploiting natural variation, and the potential of this enzyme to improve sucrose metabolism and eventually sink strength *in planta*.

## Introduction

In the plant kingdom, sucrose is the major product of photosynthesis. It is transported from photosynthetic source tissues to sink tissues as a main energy hub for metabolism, carbon source for synthesis of biomolecules, as well as an important signaling molecule [1, 2]. Therefore, the mechanism by which sucrose metabolism is regulated has direct consequences on sink strength which is defined as the competitive ability of the organ to attract the photo-assimilates [3]. As a result, sink strength plays an important role in plant growth, development, and biomass production.

In plants sucrose synthase (SuSy) is, next to invertase, a class of enzymes directly involved in hydrolysis of sucrose in sink tissues. SuSy is a member of the glycosyltransferase family, which catalyzes a reversible hydrolysis of sucrose into UDP-glucose and fructose in the presence of UDP. Up till now, six *SuSy* genes have been described in tomato (shown in S2 File). Three genes *SuSy1* (Solyc12g009300, also known as *TOMSSF* or *SUS2)*, *SuSy3* (Solyc07g042550) and *SuSy4* (Solyc09g098590) were cloned before the release of tomato genome sequence [4–6], and *SuSy5* (Solyc07g042520), *SuSy6* (Solyc03g098290) and *SuSy7* (Solyc02g081300) were identified after that [7]. These genes are shown to be expressed differentially in tomato plant tissues [4–10]. *SuSy1* is highly expressed in young fruits, whereas the expression level of *SuSy3* is highest in mature fruits. The transcript level of *SuSy4* is most abundant in sink organs such as fruits, roots, and developing leaves, but about 200-fold lower than that of *SuSy3*. Meanwhile *Susy5* is highly expressed in stems and roots, the transcript level of *Susy6* and *SuSy7* are very low in all the main tissues. At the protein level, SuSy1 and SuSy3 isoforms share up to 91% of sequence identity, while they are only 68% and 73% identical to SuSy4 and SuSy5 respectively. SuSy6 and SuSy7 isoforms are up to 73% in sequence homology, but they are only 54–56% identical to the other isoforms. These SuSy1/3/4 isoforms have been reported to be present in cytoplasm, cell membrane, and mitochondria [11–13], but little is known about the compartmentation of SuSy5/6/7. So far, SuSy1 of *Arabidopsis thaliana* is the only plant SuSy with a resolved crystal structure, which showed that this enzyme is organized in tetramers [14]. The SuSy protein structure basically contains three distinct features: i. an N-terminal domain involved in cellular compartmentation which is sub-divided into a cellular targeting domain (CTD, length 116 amino acids) and an ENOD40 peptide-binding domain (EPDB, length 120 amino acids); ii. a GT-B glycosyltransferase domain (GT-B, length 548 amino acids); and iii. a C-terminal extension of 30 up to 40 amino acids. Several residues involved in substrate binding have been identified by X-ray diffraction of enzyme crystals in complex with: i) UDP-glucose, and ii) UDP and fructose. Even though the complex of AtSuSy1 and sucrose is still missing, the residues involved in sucrose binding could be derived from the interaction with glucose and fructose of those two complexes.

An important role of SuSy on plant sink strength has been reported in tomato, where tomato plants with reduced SuSy activity, obtained by down regulating the expression of the *SuSy1* allele by an antisense approach, were shown to have a reduced fruit setting and sucrose unloading capacity in their young fruits [15]. In addition, SuSy was also shown to play an essential role in cold tolerance in chickpeas, in which significantly higher activity of SuSy was observed in seeds of the cold tolerant lines compared to that of cold susceptible ones [16]. This cold tolerance trait could be an advantage for the tomato cultivars grown in heated glasshouses during winter because it would help to reduce energy consumption under such cultivation conditions. The sub-optimal temperature that we aim for is 16°C, which is below the current optimum temperature 19–20°C for heated glasshouse tomato cultivation in the Netherlands, and well above the non-freezing temperature of 12°C which is known to cause physiological injury to tomato plants and their fruits when exposed for several weeks [17, 18]. Therefore, enhancing tomato SuSy activity is of interest to increase sink strength in order to have a positive effect on plant growth, development, and yield at optimal as well as sub-optimal temperatures. For that reason, this study focused on exploring the natural genetic variation among the three SuSy1/3/4 isoforms using the available genome sequences of 84 accessions including cultivars, landraces, and wild species from The 150 Tomato Genome ReSequencing project [19], and one extra high-altitude wild species accession obtained from Tomato Genetics Resource Center in Davis, California (TGRC, http://tgrc.ucdavis.edu/). An *in silico* analysis was carried out to predict the potential effect of amino acid changes caused by natural variation on the modelled protein structure and functionality. Furthermore, three chosen variants of SuSy1 and SuSy3 from wild accessions along with the counterparts from cultivar Moneymaker as the reference control were cloned and expressed in a heterologous system to investigate their enzyme kinetics at control (25°C) and low (16°C) temperatures.

## Materials and methods

### Computational analysis of sucrose synthase sequences from 85 tomato accessions

The genomic sequence *SuSy1/3/4* of 84 different tomato accessions, were obtained from The 150 Tomato Genome ReSequencing project, in which the raw data were submitted to the European Nucleotide Archive under project number PRJEB5235 (http://www.ebi.ac.uk/ena/data/view/PRJEB5235). Among these accessions 53 are cultivated and landrace accessions, whereas 31 are wild species accessions. In order to identify the possible coding sequences (cds) for SuSy proteins in the 84 accessions, the cds of *SuSy1* (GenBank: L19762), *SuSy3* (GenBank: AJ011319), and *SuSy4* (GenBank: HM180943) were aligned to these genomic sequences using the ClustalOmega program (http://www.ebi.ac.uk/Tools/msa/clustalo/). In addition, the cds of *SuSy1/3/*4 from the extra wild species accession *Solanum arcanum* LA385 were also included in this study. The obtained cds of 85 accessions were subsequently translated into amino acid sequences using BioEdit software v.6.0.6 (Tom Hall, Ibis Therapeutics, Carlsbad, USA).

We investigated *in silico* the effects of the amino acid substitutions within each protein sequence compared to the corresponding positions of the reference cultivar Heinz. Those studied effects included: 1) the potential impact of an amino acid change on protein function using the 'Protein Variation Effect Analyzer' program (PROVEAN) [20]; 2) the possible influence of an amino acid change on the protein secondary and tertiary structure using the 'Protein Homology/analogY Recognition Engine V 2.0' program (Phyre2) [21] and Modeller program v9.19 [22]. Phyre2 also provided the 3D-models of the SUSY variants based on the available crystal structure of SuSy1 of *Arabidopsis thaliana* (PDB entry 3S27), which were visualized with PyMol software v1.6 (The PyMOL Molecular Graphics System v1.6, Schrödinger, LLC, USA).

### Plants, bacterial and yeast strains, and plasmids used

Accessions that were used to extract DNA for sequencing and cloning of specific *SuSy* genes were: *S*. *lycopersicum* cv Moneymaker (accession number LA2706), cv Large Red Cherry (accession number TR00018), *S*. *neorickii* G1.1601 (accession number CGN24193) and *S*. *arcanum* LA385 obtained from Tomato Genetics Resource Center in Davis, California (TGRC, http://tgrc.ucdavis.edu/). The cv Moneymaker was used in this study as the reference in place of cv Heinz because they both have the same SuSy1/3/4 protein sequences. Plasmid pGEM-T Easy (Promega, USA), PCR II-Blunt-TOPO (ThermoFisher Scientific, USA) were used to construct intermediate clones, while plasmid pDR195 (a gift from Dr. Wolf Frommer—Carnegie Institution for Science, USA) was used to make the expression clones. Competent cells of *Escherichia coli* strains DH5a and XL10-Gold were purchased from Invitrogen (USA) and Agilent (USA) respectively, whereas yeast *Saccharomyces cerevisiae* strain 22574d was provided by courtesy of Dr. Bruno André—Université Libre de Bruxelles, Belgium.

### Cloning and expression of *SuSy* genes

RNA was prepared from about 5 mg fruit tissue ground in liquid nitrogen using a CTAB mini-isolation protocol [23]. The isolated RNA was then treated with DNase I (Invitrogen, USA), and converted into cDNA with iScript cDNA synthesis kit (BioRad, USA). Amplification of cDNA was achieved following a standard PCR protocol. The primer pairs for specific gene amplification can be found in S1 Table. All the amplifications for cloning steps were carried out with high fidelity PfuUltra Fusion II HS DNA polymerase (Agilent, USA). The amplified cDNA of *SuSy1*/*3/4* from cv. Moneymaker, and *SuSy1/3/4* from wild accession LA385 were ligated with plasmid pGEM-T Easy, whereas *SuSy1* from LRC and *Susy3* from wild accession G1.1601 were ligated with PCR II-Blunt-Topo plasmid. These ligation mixtures were transformed into competent *E*. *coli* DH5α and positive clones were confirmed by sequencing. Subsequently the cDNA of these genes were amplified with primer pairs containing *Bam*HI sites from the isolated plasmids, which resulted in fragment *Bam*HI-*SuSy*-*Bam*HI. The newly obtained fragment *Bam*HI-*SuSy*-*Bam*HI was digested with *Bam*HI enzyme, ligated into the *Bam*HI digested and dephosphorylated plasmid pDR195, and transformed into ultra-competent *E*. *coli* XL10-Gold (Agilent, USA). Positive clones were confirmed by restriction digestion and sequencing. The final expression plasmids were introduced into yeast *S*. *cerevisiae* strain 22574d as described in the manual of the DUALmembrane starter kit (Dualsystems Biotech AG, Switzerland). Positive clones were selected on synthetic defined (SD) medium agar without uracil (Clontech, USA).

### Cultivation of yeast

Cultivation of the transformed yeast harboring recombinant SUSY enzyme was done according to Römer et al. [24] with some modifications. Briefly, a positive colony from a SD agar plate was cultivated in a 100 mL flask containing 12 mL SD medium with 2% glucose without uracil at 30°C overnight in an incubator shaker with an orbital speed of 200 rpm. Subsequently the overnight culture was transferred into a 4 L flask containing 2 L of the same medium for 24 h incubation with an orbital speed of 130 rpm. The cells were then harvested by centrifugation at 4°C and 10,000x g and stored at -20°C till further use.

### Enzyme purification

An aliquot of 10 g harvested yeast cells were resuspended to 40% (w v^-1^) in lysis buffer of 50 mM HEPES pH 7.5, 1 mM dithiothreitol (DTT), 1 mM Na_2_EDTA, 1 mM phenylmethanesulfonyl fluoride (PMSF), 10% glycerol, 1 μM Leupeptin, and 1 μM Pepstatin A. The resuspended cells were then lysed via 3 passages of 10,000 psi using a SLM-Aminco French Pressure Cell apparatus. The cell extract was subsequently cleared by centrifugation at 18,000 x *g* for 20 min at 4°C. Afterward, the extract was purified with ion-exchange chromatography (IEX) and immobilized metal ion affinity chromatography (IMAC) according to Römer et al. [24], using HiLoad Q Sepharose high performance and HiTrap IMAC Fast Flow columns (GE Life Sciences, USA) respectively. The purification of SuSy3-haplotype#1 (cv Moneymaker) and SuSy3-haplotype#9 (wild accession LA385) were prepared by both IEX and IMAC, whereas SuSy3-haplotype#10 (wild accession G1.1601), SuSy1-haplotype#1 (cv Moneymaker), SuSy1-haplotype#3 (cv Large Red Cherry), SuSy4-haplotype#1 (cv Moneymaker), and SuSy4-haplotype#4 (wild accession LA385) were only purified with IEX. The protein concentration was measured with Pierce BCA Protein Assay Kit (ThermoFisher–Scientific, USA). Subsequently, the size of the purified products was estimated by SDS-PAGE and Native PAGE by using the Mini-PROTEAN TGX gel 4–20% (BioRad, USA). The expected product of 93 kDa in SDS PAGE was not found in the purified product of both SuSy4-haplotype#1 (cv Moneymaker) and SuSy4-haplotype#4 (wild accession LA385) with IEX (data not shown), therefore they were excluded from the enzyme kinetics analysis.

### Enzyme kinetics analysis

In this study we focused on the kinetics of the sucrose cleavage action of the enzymes under investigation. For enzyme kinetics assays, we used the highest purified enzymes that were prepared as described in section enzyme purification above, i.e. IMAC-purified SuSy3-haplotype#1 and haplotype#9, IEX-purified SuSy3-haplotype#10, SuSy1-haplotype#1, and SuSy1-haplotype#3. The kinetic constants V_max_ and K_m_ for sucrose were determined in a reaction mixture containing 20mM HEPES pH 7.5, 4 mM UDP, and sucrose ranging from 10 to 500 mM. The reaction was started by adding 0.5–1.0 μg purified SuSy enzyme into the reaction mixture and incubated at 25μC or 16μC for 0, 5, 10, and 15 min. The reaction was terminated by incubation at 95μC for 4 min. The formation of UDP-glucose was measured in an assay containing 0.4 M glycine pH 8.9, 10 mM MgCl_2_, 4mM NAD^+^, and 0.02 U of UDP-glucose dehydrogenase. The amount of NADH formed in this reaction equals the amount of UDP-glucose formed in the reaction mixtures with SuSy enzymes. For the blanks, the reaction was without UDP addition. The amounts of UDP-glucose formed in 0, 5, 10, and 15 min, corrected for the blank reactions, were plotted and fitted using a linear regression model. The slope of this line equals the initial rate of sucrose hydrolysis (ν). Subsequently, the kinetic constants V_max_ and K_m_ were calculated by fitting the Michaelis-Menten curve. We define 1 unit (U) of SuSy enzyme activity as the amount of enzyme that catalyzes the production of 1 μmol of UDP-glucose per minute. All the chemicals and UDP-glucose dehydrogenase enzyme used for kinetics analysis were purchased from Sigma-Aldrich (USA).

## Results

### More variation in the coding sequences of SuSy of wild species than in cultivars and landraces

The genomic sequences of *SuSy1/3/4* of 85 accessions were aligned against known coding sequences of *SuSy1* (GenBank: L19762), *SuSy3* (GenBank: AJ011319), and *SuSy4* (GenBank: HM180943) to search for single nucleotide polymorphisms (SNPs). We found SNPs in both coding and non-coding regions with both synonymous and non-synonymous SNPs (data not shown). For the non-synonymous SNPs that lead to amino acid substitutions, the corresponding protein sequences were deduced and grouped into 23 distinct SuSy1 sequences, 22 SuSy3 sequences, and 17 SuSy4 sequences, referred to as haplotypes. The overview of all haplotypes and the corresponding tomato accessions is presented in S2–S4 Tables. The upper part of Tables 1–3 separated by the broken line represents the haplotypes found predominantly in cultivated and landrace accessions, whereas the lower part shows the haplotypes of wild species accessions. More amino acid changes were found in protein sequences of wild species accessions than in those of cultivated and landrace accessions. The highest number of amino acid substitutions identified in wild accessions were seven, nine and five in SuSy1-haplotype#23, SuSy3-haplotype#22 and SuSy4-haplotype#17, respectively; whereas the cultivated and landrace accessions had maximally three, two and two substitutions in SuSy1-haplotype#7, SuSy3-haplotype#4, and SuSy4-haplotype#3, respectively. The identified variations in SuSy1/3/4 of 85 accessions were distributed over all domains identified for AtSuSy1: cellular target domain (CTD), ENOD40 peptide-binding domain (EPBD), GT-B glycosyltransferase domain (GT-B), and C-terminal extension (C), except for SuSy4, which had no variation in the C domain (Tables 1–3). Furthermore, the common motif E-X_7_-E, which is involved in the transfer of a glycosyl residue from a donor sugar to an acceptor, present in the glycosyltransferase family reported by Cid et al. [25], was conserved in all these tomato SuSy haplotypes (see S2 File).

**Table 1:** Tomato SuSy1 haplotypes (805aa).

Domain	CTD			EPBD		GT-B												C
Position	3	11	73	176	216	318	348	354	375	537	566	569	600	635	641	727	730	790
Ref.	E	R	S	A	I	L	G	V	R	S	K	T	G	N	R	E	K	A
Haplo.																		
1 (15)* ^$^	.	.	.	.	.	.	.	.	.	.	.	.	.	.	.	.	.	.
2	.	.	.	.	.	.	.	.	.	.	.	.	.	.	.	.	.	S
3 (5) ^$^	.	S	.	.	.	.	.	.	.	.	.	.	.	.	.	.	.	.
4 (23)	.	.	.	.	.	.	.	G	.	.	.	.	.	.	.	.	.	.
5 (4)	.	S	.	.	.	.	.	G	.	.	.	.	.	.	.	.	.	.
6 (8)	.	S	.	.	.	.	.	.	.	T	.	.	.	.	.	.	.	.
7 (12)	.	S	.	.	.	.	.	G	.	T	.	.	.	.	.	.	.	.
8	.	S	.	.	.	.	.	.	.	T	.	.	.	.	K	.	.	.
9	.	S	.	.	.	.	.	.	.	T	.	.	.	.	.	D	.	.
10	.	S	.	.	.	.	.	.	H	T	.	.	.	.	.	.	.	.
11	.	S	.	.	.	.	.	.	.	T	.	.	E	.	.	D	.	.
12	.	S	.	.	.	.	.	.	.	T	.	S	.	.	.	.	N	.
13 (3)	.	S	.	.	V	.	.	.	.	T	.	.	.	.	.	.	.	.
14	.	S	.	.	V	.	.	G	.	T	.	.	.	.	.	.	.	.
15	.	S	.	T	.	.	.	G	.	T	.	.	.	.	.	.	.	.
16	.	S	.	T	.	.	.	.	.	T	.	S	.	.	.	.	.	.
17	.	S	.	.	.	.	.	.	.	T	.	S	.	.	.	.	N	.
18	.	S	.	T	.	.	.	.	.	T	.	S	E	.	.	.	.	.
19	.	S	.	.	.	F	.	.	.	T	.	.	.	K	.	.	.	.
20	.	S	.	.	.	.	.	G	.	T	.	S	.	.	.	.	N	.
21	D	S	.	.	.	.	S	.	.	T	.	.	.	.	.	.	.	.
22	.	S	.	.	.	.	S	G	.	T	.	.	.	.	.	.	.	.
23	.	S	T	.	.	.	S	G	.	T	M	.	.	.	.	D	.	.
Effect^#^	-	-	-	-	-	+	-	+	+	-	-	-	-	-	+	-	+	-

* The number of accessions in each haplotype is given between parentheses; the haplotypes without the parenthesis means that they only have one accession.

^#^ The amino acid substitution is predicted to have (+) or not to have (-) effect on protein function.

^$^ These haplotypes are chosen to be cloned and heterogously expressed. The dot “.” indicates the same amino acid as the reference’s at the corresponding position. The broken line separates the cultivated and landrace accessions from the wild species ones above and below the line, respectively.

**Table 2:** Tomato SuSy3 haplotypes (805aa).

Domain	CTD				EPBD			GT-B												C
Position	53	73	88	106	188	199	219	349	361	546	555	559	591	604	629	634	724	727	741	798
Ref.	S	S	I	S	N	Q	S	Q	H	P	D	E	W	L	E	H	E	E	K	E
Haplo.																				
1 (50)* ^$^	.	.	.	.	.	.	.	.	.	.	.	.	.	.	.	.	.	.	.	.
2	.	.	.	.	.	.	.	.	.	.	Y	.	.	.	.	.	.	.	.	.
3 (2)	.	.	.	I	.	.	.	.	.	.	.	.	.	.	.	.	.	.	.	.
4 (8)	.	.	.	I	.	.	.	.	.	.	.	.	.	.	.	.	.	.	E	.
5	.	.	.	I	.	.	.	.	R	.	.	.	.	.	.	.	.	.	E	.
6	.	.	.	I	.	.	.	.	.	.	Y	.	.	.	.	.	.	.	E	.
7	.	.	.	I	.	.	.	.	.	.	.	.	.	.	.	.	D	.	E	.
8 (5)	.	.	.	I	.	H^a^	.	.	.	.	.	.	.	.	.	.	D	.	E	.
9 (2) ^$^	A	.	.	I	.	.	.	.	.	.	.	.	.	.	.	.	.	D	E	.
10 (4) ^$^	A	.	.	I	.	.	.	L	.	.	.	.	.	.	.	.	.	D	E	.
11	A	.	.	I	.	.	.	.	.	.	.	D	.	.	.	.	.	D	E	.
12	.	.	.	I	K	L^b^	.	.	.	.	.	.	.	.	.	.	D	D	E	.
13	.	C	.	I	K	.	.	.	.	.	.	.	.	.	.	.	D	D	E	.
14	.	C	.	I	K	.	.	.	.	.	.	.	.	.	.	.	D	.	E	.
15	.	.	F	I	.	.	.	.	.	.	.	.	.	.	.	.	D	.	E	.
16	.	.	.	I	.	.	.	.	.	S	.	.	.	.	G	.	D	.	E	.
17	A	.	.	I	.	.	.	.	.	S	.	.	.	.	G	.	D	D	E	.
18	.	.	.	I	.	.	.	.	.	S	.	.	.	.	.	.	D	D	E	K
19	.	.	.	I	K	.	.	.	.	S	.	.	G	.	.	.	D	D	E	K
20	.	.	.	I	K	H^a^	P	.	.	.	.	.	.	.	.	.	D	D	E	K
21	.	.	.	I	K	.	.	.	.	.	.	.	.	I	.	Y	D	D	E	K
22	A	.	.	I	K	H^a^	P	.	.	.	.	.	.	.	.	.	D	D	E	K
Effect^#^	-	-	+	-	-	-^a^ +^b^	-	+	-	+	-	-	+	-	-	-	-	+	-	-

* The number of accessions in each haplotype is given between parentheses; the haplotypes without the parenthesis means that they only have one accession.

^#^ The amino acid substitution is predicted to have (+) or not to have (-) effect on protein function.

^$^ These haplotypes are chosen to be cloned and heterogously expressed. The dot “.” indicates the same amino acid as the reference’s at the corresponding position. The broken line separates where the cultivated and landrace accessions from the wild species ones above and below the line, respectively. At position 199, the amino acid substation Q199H^a^ and Q199L^b^ is predicted to have neutral and deleterious on protein function, respectively.

**Table 3:** Tomato SuSy4 haplotypes (812aa).

Domain	CTD		EPBD			GT-B									
Position	35	114	175	212	229	281	325	408	567	585	634	683	688	732	738
Ref.	V	V	N	N	D	N	K	V	S	V	L	V	T	R	T
Haplo.															
1 (55)*	.	.	.	.	.	.	.	.	.	.	.	.	.	.	.
2 (5)	G	.	.	.	.	.	.	.	.	.	.	.	.	.	.
3 (1)	.	.	.	.	.	.	.	G	.	G	.	.	.	.	.
4 (5)	.	I	.	.	.	.	.	.	.	.	.	.	.	.	.
5 (2)	.	I	.	S	.	.	.	.	.	.	.	.	.	.	.
6	.	I	.	.	E	.	.	.	.	.	.	.	.	.	.
7 (2)	.	I	S	.	.	.	.	.	.	.	.	.	.	.	.
8	.	I	.	.	.	.	.	.	N	.	.	.	.	.	.
9 (2)	G	I	.	S	.	.	.	.	.	.	.	.	.	.	.
10 (2)	G	I	S	.	.	.	.	.	.	.	.	.	.	.	A
11	.	I	S	.	.	.	.	.	N	.	.	.	.	.	.
12 (2)	.	I	.	.	E	.	.	.	N	.	.	.	.	.	.
13 (2)	.	I	.	.	E	.	.	.	N	.	.	.	.	Q	.
14	.	I	.	.	E	.	.	.	N	.	.	.	I	.	.
15	.	I	.	.	E	.	.	.	N	.	.	L	I	.	.
16	.	I	.	S	.	S	.	.	.	.	F	.	.	.	.
17	G	I	K	.	.	.	N	.	N	.	.	.	.	.	.
Effect^#^	+	-	-	-	-	+	-	-	-	+	+	+	+	-	-

* The number of accessions in each haplotype is given between parentheses; the haplotypes without the parenthesis means that they only have one accession.

^#^ The amino acid substitution is predicted to have (+) or not to have (-) effect on protein function. The dot “.” indicates the same amino acid as the reference’s at the corresponding position. The broken line separates where the cultivated and landrace accessions from the wild species ones above and below the line, respectively.

### Found natural allelic variation is predicted to have no effect on protein structure

**Fig 1:**
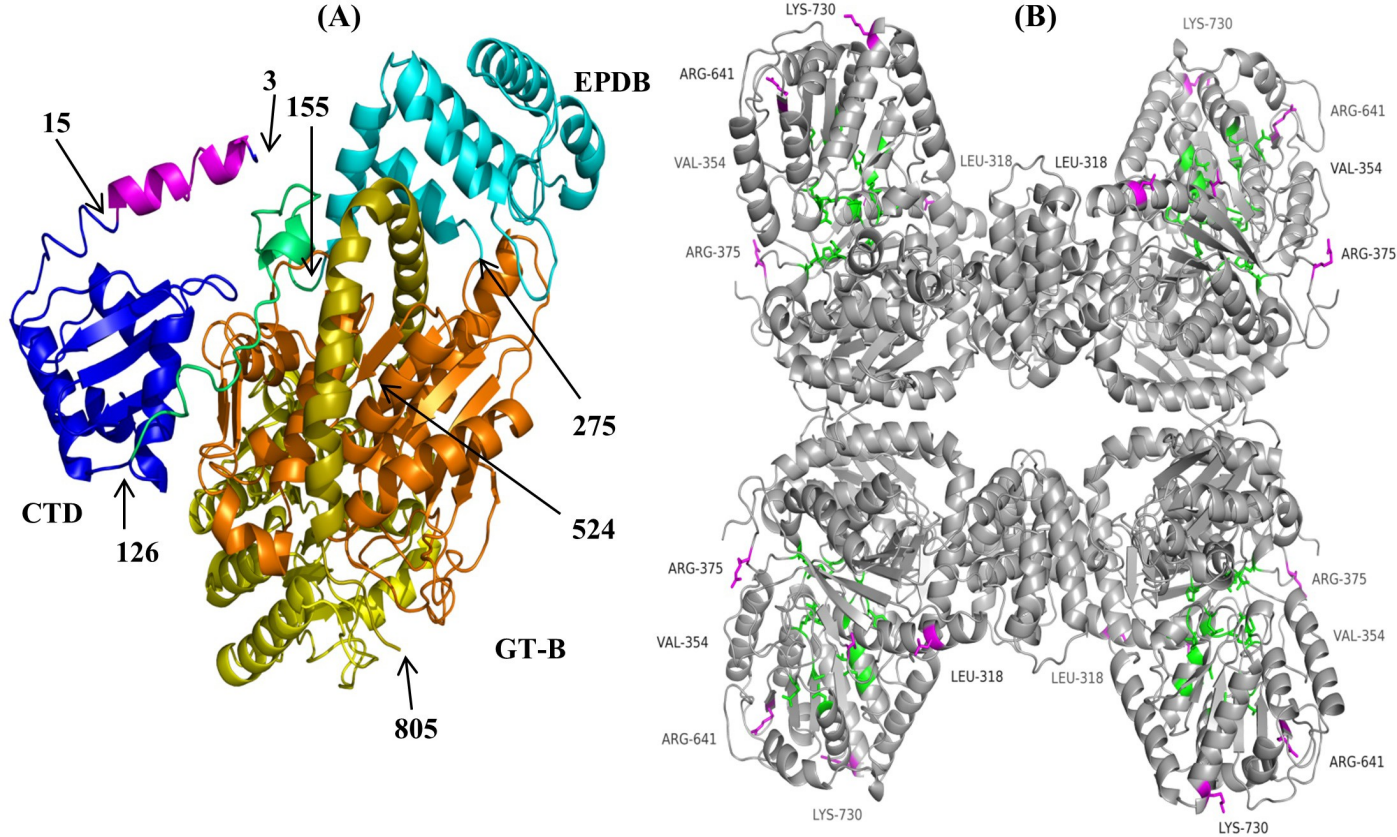
Ribbon drawings of the 3D modelled tomato SuSy1 haplotype#1 monomer and homo-tetramer. Based on the structure of AtSuSy1 (PBD ID 3S18), the monomer structure (A) starts with the cellular targeting domain colored blue (CTD, residues 3–125) containing a α1 helix (magenta, residue 3–15). A linker colored green (residues 126–154) bridges the CTD with the ENOD40 peptide-binding domain in cyan color (EPDB, residue 155–274). The structure continues with a GT-B glycosyltranferase domain (GT-B) which is divided into GT-B_N_ domain in orange (residues 275–523) and GT-B_C_ domain in yellow (residues 524–771). This monomer ends with a C-terminal extension domain (C) (residues 773–805). In the homo-tetramer model (B), the amino acid residues interacting with substrate are indicated in green, while the residues L318, V354, R375, R641, and L730 with variation in SuSy1 are highlighted in magenta. All these amino acid are not close to the binding sites, or in the interfaces between the subunits.

*A*. *thaliana* SuSy1 (PDB 3S27, 3S28 and 3S29, 808 aa) [14] is the only plant SuSy protein with the crystal structure resolved till now and thus was used as a template to generate tomato SuSy models in order to predict the effect of identified amino acid variations on the structures. A protein sequence alignment between AtSuSy1 and haplotype#1 of tomato SuSy1/3/4 shows that tomato SuSy1 and SuSy3 have the highest homology to AtSuSy1 with 78% and 77% identity respectively, while it is 68% for SuSy4 (data not shown). According to one of the criteria of template-based protein structure modeling, the sequence identity between the query (e.g. tomato SuSy) and template (e.g. AtSuSy1) should be at least 30% to obtain a model with sufficient accuracy [26]. Therefore the protein structures of all the tomato SuSy1/3/4 haplotypes are predicted with high confidence using AtSuSy1 as the template using the Phyre2 modeling suite. All the found variations in tomato SuSy1/3/4 haplotypes are predicted not to cause disruption of α-helixes or β-strands in secondary structure compared to that of the reference cultivar Heinz (data not shown). Fig 1A shows an example of a subunit of the predicted tomato SuSy1-haplotype#1 3D structure model showing 781 residues (97% of the peptide sequence). Furthermore, based on the homo-tetrameric protein structure of AtSuSy1 [14], none of the amino acid changes found in SuSys of 85 tomato accessions are predicted to be involved in subunit interactions (Figs 1B and S1 and S1 File). The corresponding residues involved in A:D subunit interaction of tomato SuSy1/3 are 129–140 and 776–794 and that of tomato SuSy4 are 132–143 and 779–799. The residues 145–152 of tomato SuSy1/3 and residues 148–155 of tomato SuSy4 of the adjacent EPDBs are involved in A:B subunit interaction.

### Substrate binding sites and phosphorylation sites are highly conserved

Functional sites of tomato SuSy proteins such as substrate binding sites and phosphorylation sites were predicted (S1 File), based on the known substrate binding sites identified in AtSuSy1 [14]. The substrate binding sites were clustered in GT-B domain and conserved in 85 tomato accessions. All the positions with amino acid variation in SuSy1/3/4 were predicted to be more than 10 Å away from the substrate binding sites and stick out to the solvent. Fig 1B shows an example of the homo-tetramer model of tomato SuSy1 displaying five residues with variation that are not in contact with the substrate binding sites and stick out to the solvent. In maize, the SuSy sites Ser-15 and Ser-170 have been reported as two conserved phosphorylation sites [13, 27]. These two sites were also conserved in all tomato SuSy3 protein variants at corresponding positions 11 and 165 and in SuSy4 at positions 11 and 168. In SuSy1, the second phosphorylation site Ser-165 was conserved in all the studied accessions, but the first phosphorylation site Ser-11 was not. This phosphorylation site Ser-11 was present in 30 out of 32 wild species accessions, whereas only less than half, 22 out of 53, cultivated and landrace accessions contained this site (Table 1).

### Several variations in SuSy are predicted to affect protein function

The potential effect of amino acid changes on protein function was also investigated *in silico*. As shown in Tables 1–3, the majority of amino acid changes was predicted to not to have an impact on protein function in the studied SuSys. However, several amino acid substitutions in each SuSy isoform were predicted to have an effect on protein function, which could be substantially lower or higher enzyme activity, or result into a profound change in enzyme kinetics. In SuSy1, variation L318F, V354G, R375H, R641K, and K730N were predicted to affect protein function, in which most of them were only found in wild species accessions, except V354G (Table 1). In SuSy3 variation I88F, Q199L, P546S, W591G, and E727D were predicted to affect protein function, and they were again only identified in wild species accessions (Table 2). In SuSy4 six variations were predicted to affect SuSy4 function, in which V35G, N281S, L634F, V683L, and T688I were found in wild species and V585G in cultivated and landrace accessions (Table 3).

### The tested SuSy3-haplotype#9 protein variant from wild accession has slightly higher sucrose-hydrolyzing capacity compared to the control from cultivated tomato

To gain insight in the role of identified natural variation on enzyme kinetics, two SuSy1 and three SuSy3 protein variants were chosen to be expressed in yeast host *S*. *cerevisiae* strain 22574d and purified for further study. The first step of enzyme purification using ion exchange chromatography (IEX) appeared to be most efficient with SuSy3 protein variants, less efficient with SuSy1 protein variants, and not successful with SuSy4 protein variants (S2 and S3 Figs). The recombinant protein variants were tested for their maximal velocity in hydrolyzing sucrose (V_max_), K_m_ for sucrose, and catalytic efficiency (V_max_/K_m_) at both 25°C and 16°C. For SuSy3, we focused on the haplotypes of two high-altitude wild accessions: *S*. *arcanum* LA385, representing haplotype#9, and *S*. *neorickii* LA0735 (also known as *S*. *neorickii* G1.1601), representing haplotype#10, collected at 2,500 and 2,900 meters above sea level respectively [28]. As shown in Table 4, low temperature had an expected impact on enzyme kinetics of all tested SuSy3 protein variants. At low temperature (16°C) the V_max_ of all three tested SuSy3 protein variants was reduced to about 55% to 75% of the values found at 25°C. The K_m_ values of these SuSy3 protein variants were about two- to three-fold higher at 16°C compared to the values found at 25°C. As a consequence, the V_max_/K_m_ values of these SuSy3 protein variants were about four-fold lower at 16°C compared to those at 25°C. SuSy3-haplotype#9 showed slightly improved kinetics with higher V_max_ and V_max_/K_m_ values at both temperatures relatively to the reference SuSy3-haplotype#1. Because SuSy3-haplotype#10 had lower purity (i.e. only purified by IEX) compared to the variants of haplotype#1 and haplotype#9 (i.e. purified by IEX and IMAC), it was not possible to make the comparison for the enzyme kinetics values between them.

**Table 4:** Enzyme kinetic parameters.

	Assay T (°C)	V_max_ (U mg ^-1^)	K_m_ (mM sucrose)	V_max_/K_m_ (U mg^-1^ μM^-1^)
SuSy1-haplotype#1 ^$^	25°C	1.2 ± 0.1	51 ± 13	24 ± 8
	16°C	0.7 ± 0.3	189 ± 90	5 ± 1 ^a^
SuSy1-haplotype#3 ^$^	25°C	1.4 ± 0.2	56 ± 14	26 ± 3
(R11S)	16°C	0.8 ± 0.2 ^a^	101 ± 15 ^a^	8 ± 3 ^b^
SuSy3-haplotype#1 ^#^	25°C	4.6 ± 0.5	34 ± 8	142 ± 38
	16°C	2.6 ± 0.4 ^b^	77 ± 22 ^a^	36 ± 7 ^b^
SuSy3-haplotype#9 ^#^	25°C	6.5 ± 1.2	33 ± 9	206 ± 50
(S53A, S106I, E727D, K741E)	16°C	3.9 ± 1.3 ^a^	75 ± 19 ^a^	52 ± 5 ^c^ *
SuSy3-haplotype#10 ^$^	25°C	6.2 ± 0.7	58 ± 14	112 ± 37
(S53A, S106I, E727D, Q349L, K741E)	16°C	4.6 ± 1.2	167 ± 29 ^a^	28 ± 6 ^a^

^$^ These proteins were purified with IEX;

^#^ These proteins were purified with IMAC. Data represent the mean of three independent measurements (± SD). Significant differences are labelled with a, b and c for *P* < 0.05, 0.01 and 0.001 between assay temperatures of each purified enzyme, respectively. The significant difference between different SuSy1/3 protein variants and the corresponding protein from the reference haplotype#1 is denoted as

* for *P* < 0.05.

### No difference in enzyme kinetics between SuSy1 protein variants with amino acid alterations at the first phosphorylation site

The effect of amino acid alterations in the first phosphorylation site of SuSy1 protein was investigated by studying enzyme kinetic parameters of SuSy1-haplotype#3 containing the phosphorylation site Ser-11 and SuSy1-haplotype#1 with Arg-11. Low temperature had the same impact on enzyme kinetics of both SuSy1 protein variants as shown in SuSy3 protein variants (Table 4). The V_max_ and K_m_ values for both SuSy1 variants was within error identical at 25°C, but the K_m_ of SuSy1-haplotype#1 increased much more compared to that of SuSy1-haplotype#3 at 16°C (i.e. 50 mM to 189 mM compared to 60 mM to 100 mM, respectively). Similarly to SuSy3 protein variants, the V_max_/K_m_ values of these SuSy1 protein variants were also about four-fold lower at 16°C compared to those at 25°C. However, there was no difference in the V_max_/K_m_ value between SuSy1-haplotype#1 and SuSy1-haplotype#3 at both temperatures.

## Discussion

### Wild accessions contain ample variation in SUSYs

The current study focused on exploring the natural genetic variation of three distinct tomato *SuSy1*, *Susy3*, and *Susy4* genes which might confer an improvement in sucrose hydrolyzing capacity. We observed the narrow genetic basis within the three *SuSy* genes of 53 cultivated and landrace accessions, whereas the number of SNPs leading to amino acid changes within SuSy1/3/4 of wild species accessions was 2.3, 4.5, and 2.5 fold higher than the most diverse counterpart from the cultivated and landrace set (Tables 1–3). This genetic erosion in cultivated tomatoes is well known, which is due to the selection for a limited number of morphological and agronomical traits such as fruit shape and size, fruit color, self-pruning, plant height and earliness [29–31]. On the other hand, the wide array of genetic diversity found in wild species is in agreement with the report from Aflitos et al. [19]. Therefore, this observed ample variation within the studied wild species could be exploited to enhance the enzyme kinetics of SuSys.

### The known active sites of SUSYs are conserved

Amino acid changes in active sites can have direct impact on enzyme kinetics. However, the *in silico* analysis showed that all the substrate binding sites found in AtSuSy1 [14] were also conserved in all three SuSy isoforms of 85 studied tomato accessions (S1 File). These active sites of SuSys are also conserved in other crops such as maize, rice, wheat, and even in non-photosynthetic bacteria *Nitrosomonas europaea* (see S1 File and [32]). This means that the residues at those active sites are crucial for enzyme activity, which was confirmed by several reports. For instance, Wu et al. [33] reported a significant loss of activity in sucrose synthesis of SuSy2 of the bacteria *Nitrosomonas europaea* when one of the conserved active site residues R567 and K575 (corresponding to R577 and K584 in tomato SuSy1/3) was altered to an Ala residue. Huang et al. [34] also observed a profound decrease of sucrose binding ability in the recombinant rice SuSy3 mutants in which three different single amino acid changes in the conserved motif E-X_7_-E were generated by site-directed mutagenesis. This conserved motif corresponded to E673-X_7_-E681 in tomato SuSy1/3. As mentioned earlier, the sucrose binding site has not been determined experimentally [14], therefore the residues involved in sucrose binding were postulated as the ones interacting with glucose and fructose moiety via the complexes AtSuSy1•UDP-glucose and AtSuSy1•UDP•fructose, respectively. These residues postulated to interact with sucrose could be confirmed by generating several inactive mutant variants of AtSuSy1, so that it can halt the fast sucrose processing. As a result, this approach could help to observe the sucrose binding to the active site in the SuSy enzyme. Several studies have successfully identified sucrose binding site by using such approach such as levansucrase of bacteria like *Bacillus substilis* and cell wall invertase of *A*. *thaliana* [35, 36].

### Recombinant SuSy3 of wild species shows an increase in sucrose hydrolyzing capacity

The aim of this study was to find the SuSy protein variants among the landrace and wild accessions having improved kinetics at different temperatures compared to that of the control cultivated tomato. Even though no variation was found in the SuSy active sites as discussed above, the potential of enhancing SuSy kinetics was seen in SuSy3-haplotype#9 of high altitude wild species *S*. *arcanum* LA385. SuSy3-haplotype#9 was able to break down sucrose more efficiently (i.e. higher catalytic efficiency V_max_/K_m_ value) than SuSy3-haplotype#1 of cv Moneymaker, especially at lower temperature of 16°C (Table 4). This accession LA385 has been reported to be less affected when grown at sub-optimal temperature of 16°C compared to two other commercial tomato cultivars, for instance less reduction of relative shoot growth rate and photosynthetic rate at both growth irradiation of 225 μmol m^-2^s^-1^ and light saturation of 1,200 μmol m^-2^s^-1^ [37]. Whether SuSy3 would play a role in better performance of this accession LA385 at sub-optimal temperature is not known because the authors Venema et al. [37] only used younger plants at vegetative stage in their study, while SuSy3 expression and SuSy activity are found to be highest in fruits [8, 38]. The authors Kortstee et al. [8] also pointed out that SuSy activity in fruits is the total sum of the activity of two main isoforms SuSy1 and SuSy3, and it does not appear to correspond to the changes in gene expression when comparing those of the commercial tomato cultivar and the wild accession LA385. It will be interesting to study the effects of the introgression of such wild SuSy3 allele into a commercial tomato on plant growth, development and yield at standard and sub-optimal growth temperatures. As for the impact of amino acid changes on protein functionality, all four amino acid changes between SuSy3-haplotype#1 and haplotype#9, as well as those found in all other SuSy protein variants in this study, are predicted to be not close to the active sites and stick out to the solvent (Figs 1 and S1). Therefore, based on protein modeling, it is still unknown how the amino acid changes in these two haplotypes could influence the catalytic efficiency. However, it has been shown that amino acid substitutions far from the active site could also drastically affect enzymatic activity [39]. Therefore experiments, such as enzyme kinetic assays, are necessary to validate the prediction of impact of amino acid changes on protein function, and to investigate the possible underlying causes of observed impacts.

### The presence of major N-terminal phosphorylation site in SuSy1 does not improve affinity for sucrose

Phosphorylation of SuSy has been reported to play an important role in its subcellular distribution and activity [40]. This study showed that SuSy1, also known as TOMSSF, from many cultivated tomato accessions lacks the major N-terminal phosphorylation site Ser-11 (Tables 2 and S2), supporting the finding of previous work [9]. On the other hand, this major phosphorylation site in SuSy1 is present amongst most of the studied wild accessions and almost half of the cultivated and landrace accessions. The result with IEX-purified SuSy1 protein variants showed that SuSy1-haplotype#3 containing Ser-11 did not show an improvement of enzyme kinetics at both temperatures compared to that of SuSy1-haplotype#1 containing Arg-11, i.e. no clear difference in V_max_, K_m_ for sucrose, and catalytic efficiency V_max_/K_m_. The phosphorylation status of the recombinant SuSy1-haplotype#3 containing the phosphorylation site Ser-11 was questionable, but the study of Sauerzapfe et al. [41] could help to answer this question. Those authors used Fe^3+^ metal affinity chromatography (Fe^3+^-IMAC) to demonstrate that their potato SuSy1 was phosphorylated when produced by the same yeast host *S*. *cerevisiae* strain 22574d as heterologous expression system and protein purification method as in our study. Those authors showed that the phosphorylated protein was eluted at higher pH compared to the dephosphorylated variant and another recombinant SuSy1 with S11A substitution. Fe^3+^-IMAC has been reported to be highly sensitive, in which one phosphate group within the protein is enough to bind to the immobilized Fe^3+^ ions and the elution towards more alkaline pH [42, 43]. At the protein level tomato SuSy1-haplotype#3 and the aforementioned potato SuSy1 are 98% identical, and both contain the conserved phosphorylation site Ser-11 (data not shown). In addition, Anguenot et al. [44] has also shown that Fe^3+^-IMAC could separate the tomato fruit SuSy non-phosphorylated protein and the phosphorylated ones. Interestingly, our results in tomato were contrasting with the findings of Sauerzapfe et al. [41] in potato, who reported that the recombinant potato SuSy1 with S11A substitution (IEX-purified) had an 8.5-fold increase in affinity for sucrose and a 300-fold decrease in V_max_ compared to that in its counterpart with phosphorylation site Ser-11 (IMAC-purified). It appears that SuSy1 kinetic properties can be impacted severely when the phosphorylation site Ser-11 is substituted to non-polar side chain residue such as Ala [41], compared to the change to positively charged residues like Arg in this study. In the same work, the authors Sauerzapfe et al. also studied the mutant SuSy1 with S11D substitution, which was to imitate the phosphorylated site, and found that its affinity to sucrose remained the same level compared to the wildtype but its V_max_ reduced significantly. This meant both substitutions S11A and S11D both drastically reduced the enzyme catalytic efficiency (V_max_/K_m_), and that phosphorylation of residue Ser-11 has a positive impact on the enzyme kinetics properties. On the other hand, in the available tetramer structures from AtSuSy1, the first 26 N-terminal amino acid residues were not resolved, thus, no structural information is available yet to examine the position of this corresponding phosphorylated residue. Further experiments are needed to shed light on this phosphorylation mechanism.

## Conclusion

Our study shows that genetic diversity in *SuSy* alleles of cultivated and landrace tomatoes is very limited, but a wide range of natural variation is found in the studied wild accessions. The enzyme kinetics study using several SuSy variants shows that SuSy3-haplotype#9 containing four amino acid changes has an enhanced catalytic efficiency compared to the reference SuSy3-haplotype#1, which is clearer at the assay temperature of 16°C than 25°C. As a result, it would be interesting to investigate the effect of introgression of such a SuSy variant with enhanced catalytic efficiency into a commercial cultivar on plant sink strength, growth, and yield at different growth temperatures. Further research is needed get better insights in the natural variation and to investigate the potential of the three extra predicted alleles *SuSy5*, *SuSy6*, and *SuSy7* to improve growth and development in tomato.

## Supporting information

S1 Fig Ribbon drawings of the 3D modelled tomato SuSy3 haplotype#1 monomer (A) and homo-tetramer (B) based on SuSy1 of A. thaliana (PDB ID 3S28). The amino acid residues interacting with substrate are indicated in green, while the residues S53, S106, Q349, E727, and K741 with variation in SuSy3-haplotype#9 and SuSy3-haplotype#10 are highlighted in magenta. All these residues with amino acid change are not close to the binding sites, or in the interfaces between the subunits.
(TIF)

S2 Fig Purification of the recombinant enzyme SuSy3-haplotype#1 (cv. Moneymaker) according to Römer et al. [24]. In the crude extract step, no specific band of expected monomer SUSY product of 92 kDa was observed in the SDS-PAGE gel (A). Once the crude extract has been purified by Ion-exchange chromatography (IEX), the expected band of approximately 90 kDa product (black arrow) became visible on SDS-PAGE gel (B). This IEX purified product went through the Immobilized metal ion affinity chromatography (IMAC) and became clearer (black arrow) with fewer unspecific band (C). The IMAC purified product was loaded on a native-PAGE and seen as the expected tetramer product (black arrow) (D). The protein ladder Precision Plus Protein Prestained Standards (BioRad, USA) was used in all the PAGE gels above.
(TIF)

S3 Fig SDS-PAGE gels of the recombinant enzymes SuSy1-haplotype#1 and SuSy4-haplotype#1 (cv. Moneymaker) purified by Ion-exchange chromatography (IEX) according to Römer et al. [24]. An enhancement of the expected monomer SuSy1-haplotype#1 product of 92 kDa (black arrow) was observed in the IEX purified sample compared to the crude extract (A). However, the enhancement of IEX-purified SuSy1-haplotype#1 was less intense when compared to that in the IEX-purified SuSy3-haplotype#1 shown in S1 Fig. For SuSy4, the expected product of 92 kDa was not found in SDS-PAGE after the purification with IEX (B). The protein ladder Precision Plus Protein Prestained Standards (BioRad, USA) was used in all the SDS-PAGE gels above.
(TIF)

S1 File Tomato *SuSy* genes information and their expression in different organs of S. lycopersicum cv Heinz based on the tomato eFP browser by R. Patel with data mode "Absolute". (XLSX)

S2 File Sequence alignment of tomato SuSy1/3/4 haplotype#1 against AtSuSy1 and SUSY from other species. (DOCX)

S1 Table Primers used for cloning. (DOCX)

S2 Table SuSy1 haplotypes and the corresponding tomato accessions. (DOCX)

S3 Table SuSy3 haplotypes and the corresponding tomato accessions. (DOCX)

S4 Table SuSy4 haplotypes and the corresponding tomato accessions. (DOCX)
