# Supplementary figures and images for: Exploring natural genetic variation in tomato sucrose synthases on the basis of increased kinetic properties
Source: PLoS One. 2018 Oct 29;13(10):e0206636. doi: 10.1371/journal.pone.0206636 (PMC6205638; doi:10.1371/journal.pone.0206636)

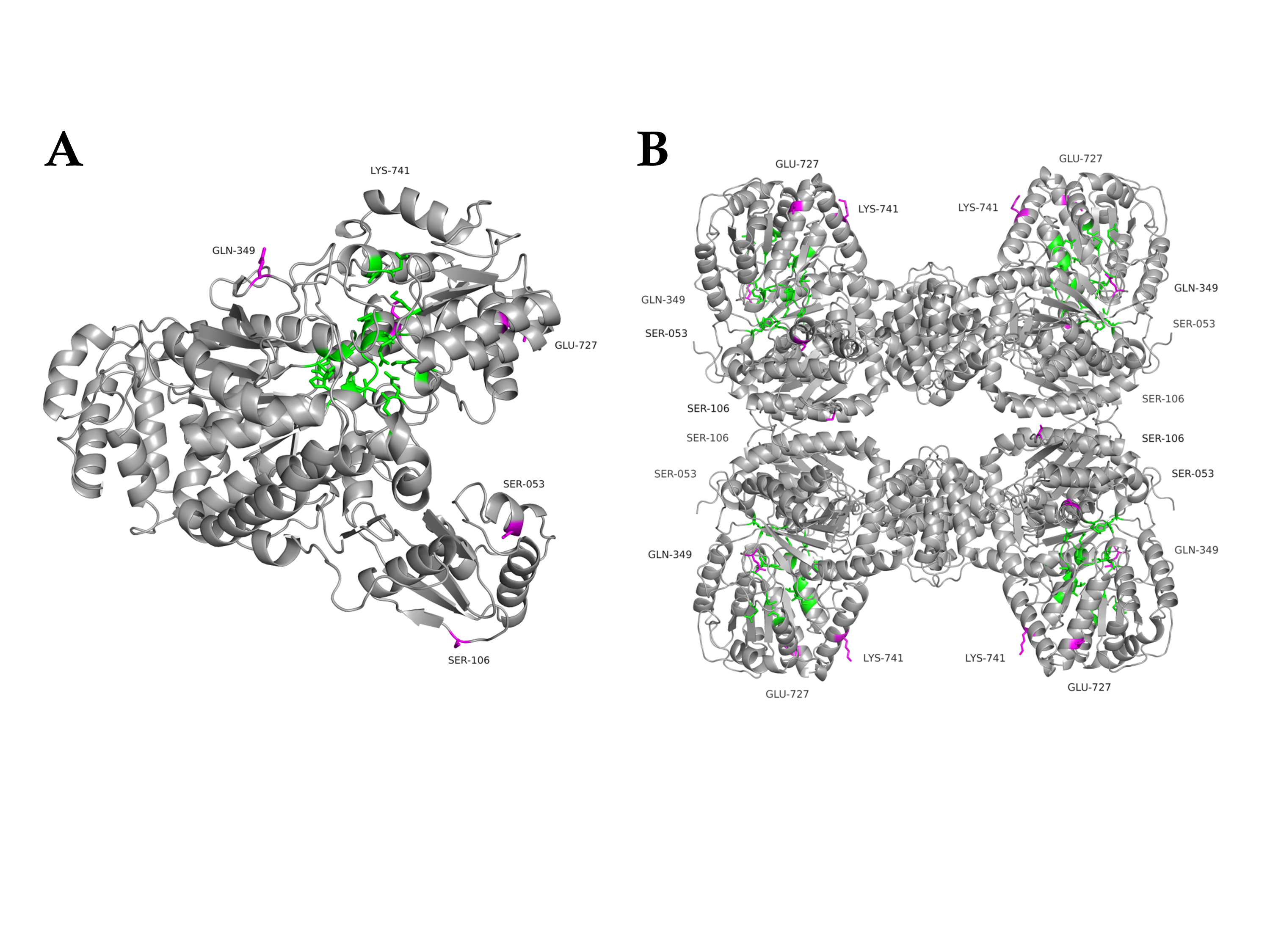

Supplement: S1 Fig — The amino acid residues interacting with substrate are indicated in green, while the residues S53, S106, Q349, E727, and K741 with variation in SuSy3-haplotype#9 and SuSy3-haplotype#10 are highlighted in magenta. All these residues with amino acid change are not close to the binding sites, or in the interfaces between the subunits. (TIF) [file pone.0206636.s001.tif]

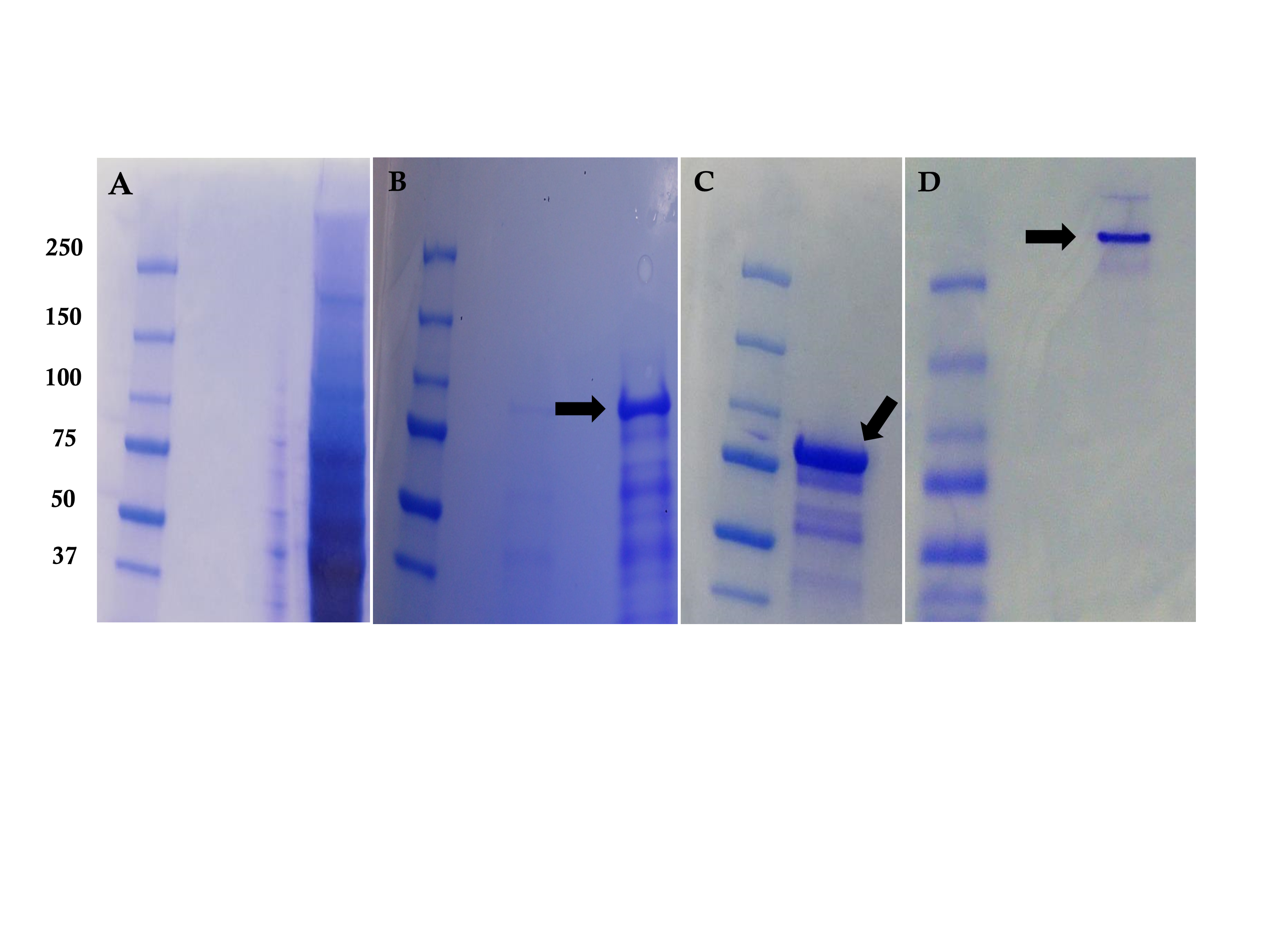

Supplement: S2 Fig — In the crude extract step, no specific band of expected monomer SUSY product of 92 kDa was observed in the SDS-PAGE gel (A). Once the crude extract has been purified by Ion-exchange chromatography (IEX), the expected band of approximately 90 kDa product (black arrow) became visible on SDS-PAGE gel (B). This IEX purified product went through the Immobilized metal ion affinity chromatography (IMAC) and became clearer (black arrow) with fewer unspecific band (C). The IMAC purified product was loaded on a native-PAGE and seen as the expected tetramer product (black arrow) (D). The protein ladder Precision Plus Protein Prestained Standards (BioRad, USA) was used in all the PAGE gels above. (TIF) [file pone.0206636.s002.tif]

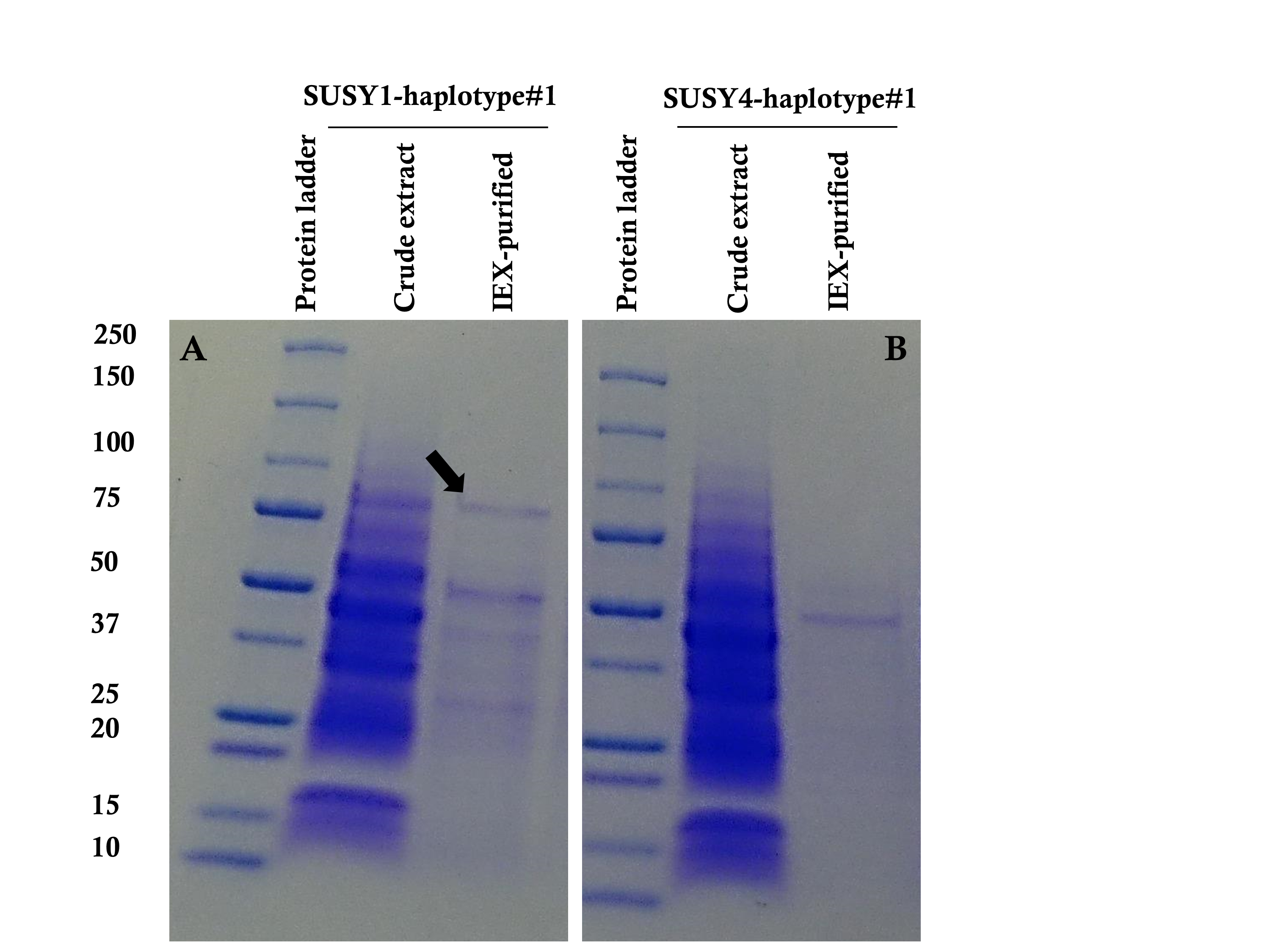

Supplement: S3 Fig — An enhancement of the expected monomer SuSy1-haplotype#1 product of 92 kDa (black arrow) was observed in the IEX purified sample compared to the crude extract (A). However, the enhancement of IEX-purified SuSy1-haplotype#1 was less intense when compared to that in the IEX-purified SuSy3-haplotype#1 shown in S1 Fig. For SuSy4, the expected product of 92 kDa was not found in SDS-PAGE after the purification with IEX (B). The protein ladder Precision Plus Protein Prestained Standards (BioRad, USA) was used in all the SDS-PAGE gels above. (TIF) [file pone.0206636.s003.tif]
