# Supplementary material for: Exploring natural genetic variation in tomato sucrose synthases on the basis of increased kinetic properties
Source: PLoS One. 2018 Oct 29;13(10):e0206636. doi: 10.1371/journal.pone.0206636 (PMC6205638; doi:10.1371/journal.pone.0206636)
Supplement: S2 File — (DOCX) [file pone.0206636.s005.docx]

**S2 File. Sequence alignment of tomato SUSY1/3/4 haplotype#1 against AtSUSY1 and SUSY from other species**

The conserved sites and motifs in the tomato SUSY1/3/4 isoforms are identified via the alignment with *Arabidopsis* SUSY1 (AtSUSY1, GenBank: OAO94355), rice SUSY3 (OsSUSY3, GenBank: ABL74561), maize SUSY1 (ZmSUSY1, GenBank: ACG43170), and SUSY2 from non-photosynthetic bacteria *Nitrosomonas europaea* (NeSUSY2, GenBank: 4RBN_A). The green, blue, red, dark red and purple lines cover different domains of SUSY as identified in AtSUSY1: cellular target domain (CTD), ENOD40 peptide-binding domain (EPBD), GT-B glycosyltransferase domain including GT-B_N_ domain and GT-B_C_ domain, and C-terminal extension (C), respectively. The cyan-highlighted residues are within 5A˚ distance from the substrates, whereas the pinkish-highlighted ones are within 10A˚ distance away from the substrates as measured with the Cn3D application (<https://www.ncbi.nlm.nih.gov/Structure/CN3D/cn3d.shtml>). The residues with small black triangles are the substrate binding sites identified in AtSUSY1 [12], which are all conserved in three tomato SUS isoforms. In addition, tomato SUS isoforms also contain the conserved E-X_7_-E motif [21] among the glycosyltransferase family enzyme, located at the residues 675-683 and highlighted with the orange line. Most of tomato SUSY also have two conserved phosphorylation sites, seen as red highlighted Ser-13 and Ser-167 in AtSUSY1. The grey-highlighted residues in tomato SUSY isoforms are the ones with natural variations among different studied accessions. For the subunit interaction found in AtSUSY1, the A:B interface was created by interaction between adjacent EPBDs and also residues 147 – 154 highlighted by the tan line. Meanwhile the A:D interface consists of the interaction between residues 131-142 and 778-796, which is highlighted by the black line.

**CTD**

AtSUSY1 --MANAERMITRVHSQRERLNETLVSERNEVLALLSRVEAKGKGILQQNQIIAEFE-ALP 57

OsSUSY3 MGETTGERALTRLHSMRERIGDSLSAHTNELVAVFSRLVNQGKGMLQPHQIIAEYNAAIP 60

ZmSUSY1 MGEGAGDRVLSRLHSVRERIGDSLSAHPNELVAVFTRLKNLGKGMLQPHQIIAEYNNAIP 60

**SlSUSY1 ----MAERVLTRVHRLRERVDATLAAHRNEILLFLSRIESHGKGILKPHELLAEFD-AIR 55**

**SlSUSY3 ----MAQRVLTRVHSLRERLDATLDAHRNEILLFLSRIESHGKGILKPHQLLAEFE-SIQ 55**

**SlSUSY4 ----MSNPKLSRIPSMRERVEDTLSAHRNQLVALLSRYVAQGKGILQPHHLIDELNNAVC 56**

NeSUSY2 ---------MTTI----DTFTTCTLQNRDAVYTLLRRYFSTNRPLLLQSDLRDELLQL-- 45

:: : : . . : : .: * .: :* .: *

AtSUSY1 EQ-TRKKLEGGPFFDLLKSTQEAIVLPPWVALAVRPRPGVWEYLRVNLHALVVEELQPAE 116

OsSUSY3 EG-EREKLKDSALEDVLRGAQEAIVIPPWIALAIRPRPGVWEYLRINVSQLGVEELSVPE 119

ZmSUSY1 EA-EREKLKDGAFEDVLRAAQEAIVIPPWVALAIRPRPGVWEYVRVNVSELAVEELRVPE 119

**SlSUSY1 QD-DKDKLNEHAFEELLKSTQEAIVLPPWVALAIRLRPGVWEYVRVNVNALVVEELSVPE 114**

**SlSUSY3 KE-DKDKLNDHAFEEVLKSTQEAIVLPPWVALAIRLRPGVWEYVRVNVNALSVEELTVPE 114**

**SlSUSY4 DDTACEKLKEGPFCEILKSTQEAIVLPPFVAIAVRPRPGVWEYVRVNVYDLSVEQLTVPE 116**

NeSUSY2 ---EKDCERSDMLHEFVFHLQEGVFSSPWAYFVLRPGIAELEFVRMHQEHLMPEKITINE 102

. . : :.: **.:. *: :.:* . *::*:: * *:: *

**A:B interface**

**A:D interface** **EPBD**

AtSUSY1 FLHFKEELVDGVKN-GNFTLELDFEPFNASIPRPTLHKYIGNGVDFLNRHLSAKLFHDKE 175

OsSUSY3 YLQFKEQLVDGSTQ-NNFVLELDFEPFNASFPRPSLSKSIGNGVQFLNRHLSSKLFHDKE 178

ZmSUSY1 YLQFKEQLVEEGPN-NNFVLELDFEPFNASFPRPSLSKSIGNGVQFLNRHLSSKLFHDKE 178

**SlSUSY1 YLQFKEELVDGASN-GNFVLELDFEPFTASFPKPTLTKSIGNGVEFLNRHLSAKMFHDKE 173**

**SlSUSY3 FLQFKEELVNGTSS-DNFVLELDFEPFTASFPKPTLTKSIGNGVEFLNRHLSAKMFHDKE 173**

**SlSUSY4 YLRFKEELVDGEDHNHLFVLELDFEPFNASVPRPSRSSSIGNGVQFLNRHLSSNMFRSNE 176**

NeSUSY2 FLGFKETVTKGEAI--ESILEVDFGPFNRAFPKLRESRSIGQGVIFLNRQLSSEMFTRIE 160

:* *** :.. **:** **. :.*: **:** ****:**:::* *

AtSUSY1 S-LLPLLKFLRLHSHQGKNLMLSEKIQNLNTLQHTLRKAEEYLAELKSETLYEEFEAKFE 234

OsSUSY3 S-MYPLLNFLRAHNYKGMTMMLNDRIRSLDALQGALRKAEKHLAGITADTPYSEFHHRFQ 237

ZmSUSY1 S-MYPLLNFLRAHNYKGMTMMLNDRIRSLSALQGALRKAEEHLSTLQADTPYSEFHHRFQ 237

**SlSUSY1 S-MAPLLEFLRAHHYKGKTMMLNDRIHNSNTLQNVLRKAEEYLIMLPPETPFFEFEHKFQ 232**

**SlSUSY3 S-MTPLLEFLRVHHYNGKSMMLNDRIQNLYTLQKVLRKAEEYLTTLSPETSYSSFEHKFQ 232**

**SlSUSY4 S-LDPLLDFLRGHNHKGNVLMLNERIQRISRLESSLNKADDYLSKLPPDTPYTDFEYALQ 235**

NeSUSY2 AGSTRLLHFLGVHTIDGQQLMFTSNSHNINMVRSQLRQALEMLEAVDGTTPWAELSSDMS 220

: **.** * .* :*:... : :. *.:* . * : * : .: :.

**GT-B_N_**

AtSUSY1 EIGLERGWGDNAERVLDMIRLLLDLLEAPDPCTLETFLGRVPMVFNVVILSPHGYFAQDN 294

OsSUSY3 ELGLEKGWGDCAQRVRETIHLLLDLLEAPEPSALEKFLGTIPMVFNVVILSPHGYFAQAN 297

ZmSUSY1 ELGLEKGWGDCAKRAQETIHLLLDLLEAPDPSTLEKFLGTIPMVFNVVILSPHGYFAQAN 297

**SlSUSY1 EIGLEKGWGDTAERVLEMVCMLLDLLEAPDSCTLEKFLGRIPMVFNVVILSPHGYFAQEN 292**

**SlSUSY3 EIGLERGWGDTAERVLEMICMLLDLLEAPDSCTLEKFLSRIPMVFNVVILSPHGYFAQEN 292**

**SlSUSY4 EMGFEKGWGDTANRVLETMHLLSDILQAPDPSTLETFLGRLPMVFNVVILSPHGYFGQAN 295**

NeSUSY2 KIGFAPGWGHNAARVAETMNMLMDILEAPSPSALEAFLARIPMISRLLILSPHGYFGQDN 280

::*: ***. * *. : : :* *:*:**. .:** **. :**: .::********.* *

AtSUSY1 VLGYPDTGGQVVYILDQVRALEIEMLQRIKQQGLNIKPRILILTRLLPDAVGTTCGERLE 354

OsSUSY3 VLGYPDTGGQVVYILDQVRAMENEMLLRIKQQGLNITPRILIVTRLLPDAHGTTCGQRLE 357

ZmSUSY1 VLGYPDTGGQVVYILDQVRAMENEMLLRIKQCGLDITPKILIVTRLLPDATGTTCGQRLE 357

**SlSUSY1 VLGYPDTGGQVVYILDQVPALEREMLKRIKEQGLDIIPRILIVTRLLPDAVGTTCGQRLE 352**

**SlSUSY3 VLGYPDTGGQVVYILDQVPALEREMLKRIKEQGLDIKPRILIVTRLLPDAVGTTCGQRLE 352**

**SlSUSY4 VLGLPDTGGQVVYILDQVRALEAEMLLRIKQQGLNFKPRILVVTRLIPDAKGTTCNQRLE 355**

NeSUSY2 VLGLPDTGGQVVYILDQVRALEQEMRDRLQLQGVQVEPKILIVTRLIPDAGDTTCNQRLE 340

*** ************** *:* ** *:: *::. *:**::***:*** .***.:***

AtSUSY1 RVYDSEYCDILRVPFRTEKG-IVRKWISRFEVWPYLETYTEDAAVELSKELNGKPDLIIG 413

OsSUSY3 KVLGTEHTHILRVPFRTENG-TVRKWISRFEVWPYLETYTDDVAHEISGELQATPDLIIG 416

ZmSUSY1 KVLGTEHCHILRVPFRTENG-IVRKWISRFEVWPYLETYTDDVAHEIAGELQANPDLIIG 416

**SlSUSY1 KVYGTEHSHILRVPFRTEKG-IVRKWISRFEVWPYMETFIEDVAKEISAELQAKPDLIIG 411**

**SlSUSY3 KVFGTEHSHILRVPFRTEKG-IVRKWISRFEVWPYMETFIEDVGKEITAELQAKPDLIIG 411**

**SlSUSY4 RISGTEYSHILRVPFRTENG-ILHKWISRFDVWPYLEKFTEDVAGEMSAELQGVPDLIIG 414**

NeSUSY2 KVSGCTNTWILRVPFRKKNGEIIPQWISRFEIWPHLETFALDVEREALAELGRRPDLIIG 400

:: . *******.::* : :*****::**::*.: *. * ** ******

AtSUSY1 NYSDGNLVASLLAHKLGVTQCTIAHALEKTKYPDSDIYWKKLDDKYHFSCQFTADIFAMN 473

OsSUSY3 NYSDGNLVACLLAHKLGVTHCTIAHALEKTKYPNSDLYWKKFEDHYHFSCQFTADLIAMN 476

ZmSUSY1 NYSDGNLVACLLAHKMGVTHCTIAHALEKTKYPNSDLYWKKFEDHYHFSCQFTTDLIAMN 476

**SlSUSY1 NYSEGNLAASLLAHKLGVTQCTIAHALEKTKYPDSDIYWKKFDEKYHFSSQFTADLIAMN 471**

**SlSUSY3 NYSEGNLAASLLAHKLGVTQCTIAHALEKTKYPDSDIYLNKFDEKYHFSAQFTADLIAMN 471**

**SlSUSY4 NYSDGNLVASLLAYKMGITQCTIAHALEKTKYPDSDIYWKKFEEKYHFSCQFTADLLSMN 474**

NeSUSY2 NYSDGNLVATLLSRRLGVTQCNIAHALEKTKYLHSDIYWQENEDKYHFSCQYTADLLAMN 460

***:***.* **: ::*:*:*.********** .**:* :: :::****.*:*:*:::**

**GT-B_C_**

AtSUSY1 HTDFIITSTFQEIAGSKETVGQYESHTAFTLPGLYRVVHGIDVFDPKFNIVSPGADMSIY 533

OsSUSY3 HADFIITSTFQEIAGNKETVGQYESHMAFTMPGLYRVVHGIDVFDPKFNIVSPGADMSIY 536

ZmSUSY1 HADFIITSTFQEIAGNKDTVGQYESHMAFTMPGLYRVVHGIDVFDPKFNIVSPGADLSIY 536

**SlSUSY1 HTDFIITSTFQEIAGSKDTVGQYESHMAFTMPGLYRVVHGINVFDPKFNIVSPGADINLY 531**

**SlSUSY3 HTDFIITSTFQEIAGSKDTVGQYESHMAFTMPGLYRVVHGIDVFDPKFNIVSPGADVNLY 531**

**SlSUSY4 HSDFIITSTYQEIAGTKNTVGQYESHTAFTLPGLYRVVHGIDVFDPKFNIVSPGADMTIY 534**

NeSUSY2 AADFIVTSTYQEIAGTREAEGQYESYRAFSMPGLYRVINGIDLFDPKFNIVSPGADAEVY 520

:***:***:*****.::: *****: **::******::**::************* :*

AtSUSY1 FPYTEEKRRLTKFHSEIEELLYSDVENKEHLCVLKDKKKPILFTMARLDRVKNLSGLVEW 593

OsSUSY3 FPFTESQKRLTSLHLEIEELLFSDVENTEHKFVLKDKKKPIIFSMARLDHVKNLTGLVEL 596

ZmSUSY1 FPYTESHKRLTSLHPEIEELLYSQTENTEHKFVLNDRNKPIIFSMARLDRVKNLTGLVEL 596

**SlSUSY1 FPYSESEKRLTAFHPEIDELLYSDVENDEHLCVLKDRTKPILFTMARLDRVKNLTGLVEW 591**

**SlSUSY3 FPYSEKEKRLTTFHPEIEDLLFSDVENEEHLCVLKDRNKPIIFTMARLDRVKNLTGLVEW 591**

**SlSUSY4 FPYFDKEKRLTSLHPSIEKLLFDPEQNEVHIGSLNDQSKPIIFSMARLDRVKNITGLVEC 594**

NeSUSY2 FPYTDQSRRLHSLIPEIESMLFDNTANFPARGILQDSDKPLIFTMARLDRIKNITGLVES 580

**: :. :** : .*:.:*:. * *:* **::*:*****::**::****

AtSUSY1 YGKNTRLRELANLVVVGGDR-RKESKDNEEKAEMKKMYDLIEEYKLNGQFRWISSQMDRV 652

OsSUSY3 YGRNPRLQELVNLVVVCGDH-GKESKDKEEQAEFKKMFNLIEQYNLNGHIRWISAQMNRV 655

ZmSUSY1 YGRNKRLQELVNLVVVCGDH-GNPSKDKEEQAEFKKMFDLIEQYNLNGHIRWISAQMNRV 655

**SlSUSY1 YAKNPRLRGLVNLVVVGGDR-RKESKDLEEQAEMKKMYELIETHNLNGQFRWISSQMNRV 650**

**SlSUSY3 YAKNPRLRELVNLVVVGGDR-RKESKDLEEQAEMKKMYELIKTHNLNGQFRWISSQMNRV 650**

**SlSUSY4 YAKNATLRELANLVVVAGYNDVKKSNDREEIAEIEKMHALMKEHNLDGQFRWISAQMNRA 654**

NeSUSY2 YGASQRLRSLANLVIVGGKIDPQHSSDHEEQEQIHQMHHLMDEYKLDPQVRWLGMRLDKN 640

*. . *: *.***:* * : *.* ** ::.:*. *:. ::*: :.**:. ::::

AtSUSY1 RNGELYRYICDTKGAFVQPALYEAFGLTVVEAMTCGLPTFATCKGGPAEIIVHGKSGFHI 712

OsSUSY3 RNGELYRYICDMRGAFVQPALYEAFGLTVIEAMTCGLPTFATAYGGPAEIIVHGVSGYHI 715

ZmSUSY1 RNGELYRYICDTKGAFVQPAFYEAFGLTVVEAMTCGLPTFATAYGGPAEIIVHGVSGYHI 715

**SlSUSY1 RNGELYRYIADTKGAFVQPAFYEAFGLTVVEAMTCGLPTFATNHGGPAEIIVHGKSGFHI 710**

**SlSUSY3 RNGELYRYIADTRGAFVQPAFYEAFGLTVVEAMSCGLPTFATNQGGPAEIIVHGKSGFQI 710**

**SlSUSY4 RNGELYRYIADKRGIFVQPAYYEAFGLTVVEAMTCGLPTFATCHGGPMEIIQDGVSGYHI 714**

NeSUSY2 LAGELYRYIADKRGIFVQPALFEAFGLTIIEAMASGLPTFATRYGGPLEIIQHNRSGFHI 700

*******.* :* ***** :******::***:.******* *** *** .. **::*

AtSUSY1 DPYHGDQAADTLADFFTKCKEDPSHWDEISKGGLQRIEEKYTWQIYSQRLLTLTGVYGFW 772

OsSUSY3 DPYQNDKASALLVEFFEKCQEDPNHWIKISQGGLQRIEEKYTWKLYSERLMTLSGVYGFW 775

ZmSUSY1 DPYQGDKASALLVDFFDKCQAEPSHWSKISQGGLQRIEEKYTWKLYSERLMTLTGVYGFW 775

**SlSUSY1 DPYHGEQAADLLADFFEKCKKEPSHWETISTGGLKRIQEKYTWQIYSERLLTLAAVYGFW 770**

**SlSUSY3 DPYHGEQAADLLAEFFEKCKVDPSHWEAISKGGLKRIQEKYTWQIYSDRLLTLAAVYGFW 770**

**SlSUSY4 DPYHPNKAAELMVEFFQRCEQNPTHWENISASGLQRILDRYTWKIYSERLMTLAGVYGFW 774**

NeSUSY2 DPNQGTATADLIADFLEKSHEKPLEWERLSQGALARVASRYTWKLYAERMMTLSRIYSFW 760

** : :: :.:*: :.. .* .* :* ..* *: .:***::*::*::**: :*.**

**A:D interface**

**C**

AtSUSY1 KHVSNLDRLEARRYLEMFYALKYRPLAQAVPLAQDD----- 808

OsSUSY3 KYVTNLDRRETRRYLEMLYALKYRKMATTVPLAIEGEASTK 816

ZmSUSY1 KYVSNLERRETRRYLEMLYALKYRTMASTVPVAVEGEPSSK 816

**SlSUSY1 KHVSKLDRLEIRRYLEMFYALKYRKMAEAVPLAAE*----- 805**

**SlSUSY3 KHVSKLDRLEIRRYLEMFYALKFRKLAELVPLAVE------ 805**

**SlSUSY4 KLVSKLERRETRRYLEMFYILKFRELVKSVPLAVDEKQ--- 812**

NeSUSY2 KFVSGLEREETDLYLNMFYHLQFRPLANRLAGDA------- 794

* *: *:* * **:*:* *::* :. :
