# Supplementary material for: Exploring natural genetic variation in tomato sucrose synthases on the basis of increased kinetic properties
Source: PLoS One. 2018 Oct 29;13(10):e0206636. doi: 10.1371/journal.pone.0206636 (PMC6205638; doi:10.1371/journal.pone.0206636)
Supplement: S1 Table — (DOCX) [file pone.0206636.s006.docx]

**S1 Table. Primers used for cloning**

| Primer name | Primer sequence (5’ – 3’) |
| --- | --- |
| SUSY1_start_fw | ATGGCTGAACGTGTTCTGACTCG |
| SUSY1_end_rv | TCACTCAGCAGCCAATGGAACAG |
| SUSY1_BamHI_fw | CGC**GGATCC**ATGGCTGAACGTGTTCTGACTCG |
| SUSY1_BamHI_rv | GCT**GGATCC**TCACTCAGCAGCCAATGGAACAG |
| SUSY3_start_fw | ATGGCTCAACGTGTTCTAACTCG |
| SUSY3_end_rv | TTACTCAACAGCCAATGGGACAA |
| SUSY3_BamHI_Fw | CGC**GGATCC**ATGGCTCAACGTGTTCTAACTCG |
| SUSY3_BamHI_Rv | GAT**GGATCC**TTACTCAACAGCCAATGGGACAA |
| SUSY4_start_fw | ATGTCGAATCCAAAGTTGTCAAG |
| SUSY4_end_rv | TCACTGCTTCTCATCAACTGCTA |
| SUSY4_BamHI_Fw | CGCATA**GGATCC**ATGTCGAATCCAAAGTTGTCAAG |
| SUSY4_BamHI_Rv | GGCATC**GGATCC**TCACTGCTTCTCATCAACTGCTA |
