# Supplementary material for: Exploring natural genetic variation in tomato sucrose synthases on the basis of increased kinetic properties
Source: PLoS One. 2018 Oct 29;13(10):e0206636. doi: 10.1371/journal.pone.0206636 (PMC6205638; doi:10.1371/journal.pone.0206636)
Supplement: S3 Table — (DOCX) [file pone.0206636.s008.docx]

**S3 Table. SUSY3 haplotypes and the corresponding tomato accessions**

| Haplotype | Variations* | Accessions** |
| --- | --- | --- |
| 1 | none | S.lyc LA2706 S.lyc LA2838A S.lyc PI406760 S.lyc LA1090 S.lyc EA00325  S.lyc EA00488 S.lyc EA00375 S.lyc EA00371 S.lyc LA2463 S.lyc LYC1969  S.lyc LYC1738 S.lyc LYC3476 S.lyc TR00003 S.lyc EA01155 S.lyc EA01049  S.lyc LYC3153 S.lyc EA03222 S.lyc PI129097 S.lyc PI272654 S.lyc EA00990  S.lyc EA00157 S.lyc EA02054 S.lyc PI303721 S.lyc LA4451 S.lyc V710029  S.lyc PC11029 S.lyc PI93302 S.lyc SG16 S.lyc EA01088 S.lyc PI203232  S.lyc PI311117 S.lyc LA1324 S.lyc PI158760 S.lyc LYC1410 S.lyc PI169588  S.lyc TR00018 S.lyc EA00940 S.lyc TR00019 S.lyc EA01019 S.lyc TR00020  S.lyc EA01037 S.lyc TR00021 S.lyc TR00022 S.lyc TR00023 S.lyc EA01640  S.lyc LA4133 S.pim LYC2740 S.lyc LYC2910 |
| 2 | D555Y | S.che LA1401 |
| 3 | S106I | S.che LA0483 S.gal LA1044 |
| 4 | S106I, K741E | S.lyc LYC1343 S.lyc LYC3306 S.lyc LA0113 S.lyc LYC2962 S.lyc LA1421  S.lyc LA1479 S.pim LYC2798 S.lyc CGN15820 |
| 5 | S106I, H361R, K741E | S.pim LA1584 |
| 6 | S106I, D555Y, K741E | S.pim LA1578 |
| 7 | S106I, E724D, K741E | S.hab LA1718 |
| 8 | S106I, Q199H, E724D, K741E | S.hab CGN157591 S.hab CGN157592 S.hab LA1777  S.hab LA0407 S.hab LYC4 |
| 9 | S53A, S106I, E727D, K741E | S.arc LA385 S.arc LA2172 |
| 10 | S53A, S106I, Q349L, E727D, K741E | S.chm LA2663 S.chm LA2695 S.neo LA2133  S.neo LA0735 |
| 11 | S53A, S106I, E559D, E727D, K741E | S.arc LA2157 |
| 12 | S106I, N188K, Q199L, E724D, E727D, K741E | S.cor LA0118 |
| 13 | S73C, S106I, N188K, E724D, E727D, K741E | S.per LA1278 |
| 14 | S73C, S106I, N188K, E724D, K741E | S.per LA1954 |
| 15 | I88F, S106I, E724D, K741E | S.hab PI134418 |
| 16 | S106I, P546S, E629G, E724D, K741E | S.pen LA0716 |
| 17 | S53A, S106I, E724D, E727D, K741E | S.pen LA1272 |
| 18 | S106I, E724D, E727D, K741E, E798K | S.hua LA1983 |
| 19 | S106I, N188K, W591G, E724D, E727D, K741E,  E798K | S.chi CGN15530 |
| 20 | S106I, N188K, Q199H, S219P, E724D, E727D,  K741E, E798K | S.hua LA1365 |
| 21 | S106I, N188K, H634Y, E724D, E727D, K741E,  E798K | S.chi CGN15532 |
| 22 | S53A, S106I, N188K, Q199H, S219P, E724D,  E727D, K741E, E798K | S.hua LA1364 |

* x#y: x, amino acid of the reference Heinz cultivar; #, amino acid position; y, amino acid of the variant

** S, Solanum; lyc, lycopersicum; cor, corneliomulleri; pim, pimpinellifolium; neo, neorickii; hua, huaylasense; hab, habrochaites; pen, pennellii; chm, chiemliewskii; chi, chilense; arc, arcanum; che, cheesmaniae; gal, galapagense. The accessions in black letters are listed as (old) cultivars and landraces, whereas the ones in red letters are considered as wild species.
