# Supplementary material for: Exploring natural genetic variation in tomato sucrose synthases on the basis of increased kinetic properties
Source: PLoS One. 2018 Oct 29;13(10):e0206636. doi: 10.1371/journal.pone.0206636 (PMC6205638; doi:10.1371/journal.pone.0206636)
Supplement: S4 Table — (DOCX) [file pone.0206636.s009.docx]

**S4 Table. SUSY4 haplotypes and the corresponding tomato accessions**

| Haplotype | Variations* | Accessions** |
| --- | --- | --- |
| 1 | None | S.lyc LA2706 S.lyc LA2838A S.lyc PI406760 S.lyc LA1090 S.lyc EA00325  S.lyc EA00488 S.lyc EA00375 S.lyc EA00371 S.lyc LA2463 S.lyc LYC1969  S.lyc LYC1738 S.lyc LYC3476 S.lyc TR00003 S.lyc LYC1343 S.lyc LYC3306  S.lyc EA01155 S.lyc EA01049 S.lyc LYC3153 S.lyc EA03222 S.lyc PI129097  S.lyc PI272654 S.lyc EA00990 S.lyc EA00157 S.lyc EA02054 S.lyc PI303721  S.lyc LA4451 S.lyc V710029 S.lyc PC11029 S.lyc PI93302 S.lyc SG16  S.lyc EA01088 S.lyc PI203232 S.lyc PI311117 S.lyc LA1324 S.lyc PI158760  S.lyc LA0113 S.lyc LYC1410 S.lyc PI169588 S.lyc TR00018 S.lyc EA00940  S.lyc TR00019 S.lyc EA01019 S.lyc TR00020 S.lyc EA01037 S.lyc TR00022  S.lyc EA01640 S.lyc LA4133 S.lyc LA1421 S.pim LYC2740 S.che LA1401  S.lyc LYC2910 S.pim LYC2798 S.che LA0483 S.pim LA1578 S.gal LA1044 |
| 2 | V35G | S.lyc LYC2962 S.lyc TR00021 S.lyc LA1479 S.pim LA1584 S.lyc CGN15820 |
| 3 | V408G, V585G | S.lyc TR00023 |
| 4 | V114I | S.arc LA385 S.per LA1954 S.hua LA1983 S.chi CGN15532 S.chi CGN15530 |
| 5 | V114I, N212S | S.hua LA1365 S.pen LA1272 |
| 6 | V114I, D229E | S.hab LA1777 |
| 7 | V114I, N175S | S.chm LA2663 S.chm LA2695 |
| 8 | V114I, S567N | S.arc LA2157 |
| 9 | V35G, V114I, N212S | S.per LA1278 S.hua LA1364 |
| 10 | V35G, V114I, N175S, T738A | S.neo LA2133 S.neo LA0735 |
| 11 | V114I, N175S, S567N | S.arc LA2172 |
| 12 | V114I, D229E, S567N | S.hab PI134418 S.hab LA1718 |
| 13 | V114I, D229E, S567N, R732Q | S.hab CGN157591 S.hab LYC4 |
| 14 | V114I, D229E, S567N, T688I | S.hab CGN157592 |
| 15 | V114I, D229E, S567N, V683L, T688I | S.hab LA0407 |
| 16 | V114I, N212S, N281S, L634F | S.cor LA0118 |
| 17 | V35G, V114I, N175K, K325N, S567N | S.pen LA0716 |

* x#y: x, amino acid of the reference Heinz cultivar; #, amino acid position; y, amino acid of the variant

** S, Solanum; lyc, lycopersicum; cor, corneliomulleri; pim, pimpinellifolium; neo, neorickii; hua, huaylasense; hab, habrochaites; pen, pennellii; chm, chiemliewskii; chi, chilense; arc, arcanum; che, cheesmaniae; gal, galapagense. The accessions in black letters are listed as (old) cultivars and landraces, whereas the ones in red letters are considered as wild species
